# Application of the Modified Basic Life Support Training Model in Improving Community Residents' Rescue Willingness in Nantong City in China

**DOI:** 10.1155/2022/6702146

**Published:** 2022-12-12

**Authors:** Yu-Fei Qian, Yu-Qin Ren, Li Wang, Rong-Qian Sun, Dan-Feng Li

**Affiliations:** Department of Emergency, The Second Affiliated Hospital of Nantong University, Nantong 226006, China

## Abstract

**Objective:**

This study explores the application and effect of the modified basic life support training in improving the first-aid level and rescue willingness of community residents in China.

**Methods:**

A total of 94 residents of a community in Nantong city were selected as the subjects by cluster sampling to receive the modified basic life support (BLS) training. The BLS knowledge, attitudes, and behaviors of all recruited subjects were evaluated by a questionnaire before and after training. A skill operation assessment was used to evaluate the effectiveness of the modified BLS training.

**Results:**

There were statistically significant differences in the BLS rescue willingness, theory, and skill scores before and after the training (*P* < 0.01). A total of 93.62% of the residents considered the modified BLS training model easier to learn and acceptable than the traditional model, and 92.55% of them thought the training content and teaching arrangement were reasonable.

**Conclusion:**

The modified BLS training model could improve the community residents' rescue willingness and skill mastery rates, enhance their first-aid skills and awareness, reduce the risk of disease transmission to a certain extent, and improve the success rate of prehospital first aid to ensure the safety of rescuers and patients.

## 1. Introduction

The first witness, namely the first responder, refers to the first person to witness an event or arrive at the scene [1]. The “first witness” can be any resident and is not specifically a medical worker. Cardiac arrest (CA) is the leading cause of death in China's residents [2], and 70%–80% of CA events occur outside a hospital [3]. Basic life support (BLS) is the primary measure to rescue patients with CA, and their survival rate decreases by 7%–10% for every 1 min delay in the intervention [4]. The survival rate of out-of-hospital patients with CA can be greatly improved if the first witness can master BLS techniques. The study by Liu and Jin [5] recruited 1212 Chinese participants and found that 55.2% of them had learned CPR, which was higher than the average CPR training rate in China in 2011 (25.6%). However, the cognition, implementation ability, and willingness of Chinese residents to initiate on-site first aid remain at low levels [6, 7]. The existing investigations show that legal problems, lack of technology, and lack of confidence constitute the main factors affecting residents' rescue efforts [5, 8, 9]. Meanwhile, there is a lack of unified and standardized BLS teaching curriculums and models in China, while the “practice while watching” (PWW) teaching method proposed by the American Heart Association (AHA) is a representative of standardized training [10]. The modified BLS training model is based on the 2020 AHA Guidelines for Cardiopulmonary Resuscitation and Cardiovascular Emergencies, which improves the traditional training model by addressing the above issues, making the BLS training more accessible and acceptable to the general population, without violating the principles of CPR. In this study, we aimed to explore the application of the modified BLS training in improving the first-aid level and rescue willingness of community residents in Nantong city in China.

## 2. Study Methods

### 2.1. Subjects

The residents of a community in Nantong city were selected from June 2021 to July 2021 as the study subjects by cluster sampling. Inclusion criteria were as follows: ① residents aged ≥18 years who volunteered to participate in the study; ② healthy residents who could perform cardiopulmonary resuscitation (CPR); ③ residents who could communicate and understand words; ④ nonmedical professionals. Exclusion criteria were as follows: ① those who could not learn due to serious audio-visual impairment; ② those who could not perform CPR due to obvious physical dysfunction or poor health conditions. The whole-group random sampling method was used to enroll participants. Residents who met the criteria of enrollment were asked whether they were willing to participate in the study and the enrollment was on a voluntary basis. The Ethics Committee of Nantong First People's Hospital approved this study, ethics no. 2020KT066.

### 2.2. Sample Size Calculation

The following formula was used to calculate sample size: *n*=[(*μ*_*α*_+*μ*_*β*_)*α*/*δ*]2 as previously described [8], where *α* = 0.05, *β* = 0.1. The mean of the total questionnaire score of residents was 31.28 ± 8.45 points. Considering lost visits and midway withdrawal and that the sample size was expanded by 20%, the sample size was calculated as 80. The total number of recruited subjects was 94 cases, which met the sample size requirement.

### 2.3. Questionnaire

The self-designed questionnaire for Basic Life Support Survey of Community Residents [11] prepared using the Delphi method was used in this study, with Cronbach's *α* coefficient of internal consistency of 0.719 and the retest reliability of 0.707. The questionnaire mainly consisted of four parts, including the general circumstances of the subjects and three subquestionnaires about their BLS knowledge, attitudes, and behaviors. The value assignment for “Yes or No” questions: 2 scores for “Yes” and 0 scores for “No;” questions on attitude: the Likert 3 score scale was used: 1 to 3 scores for “unwilling,” “uncertain,” and “very willing,” respectively; multiple-choice questions: 2 scores for correct answers and 0 scores for wrong answers; multiple answer questions: 2 scores for correct answers, 1 score for incomplete answers, and 0 score for incorrect answers. The questionnaire survey was conducted before and after the training, and the trained staff explained the survey before instruction without guiding language. The questionnaire was filled in within 6–12 min to ensure its validity.

### 2.4. Basic Life Support Operation Evaluation Form

The adult BLS single and double operation evaluation forms of the AHA Training Center were used.

### 2.5. Satisfaction Survey

The self-designed questionnaire was used for the satisfaction survey. The questionnaire contents included the community residents' views on whether the teaching content and course arrangement of the modified BLS training model were reasonable, it was easy, it was in line with the residents' learning needs, and it was easier for residents to grasp the content. The scores were given using the Likert 5 score scale, with the satisfaction (%) = ([number of “very satisfied” + “satisfied”]/total number of people) × 100%.

### 2.6. Training Outline

The training outline was developed based on the AHA Guidelines for Cardiopulmonary Resuscitation and Cardiovascular First Aid in 2020 [4] (guidelines in 2020). The investigation and research [8, 12–15] found that fear of legal problems, lack of knowledge, fear of disease transmission, and other factors constituted the main reasons for reluctance to assist in emergencies. The modified BLS training model improved the traditional training model in terms of the above factors. It focuses on the role of the first witness in emergency situations, how to call for help, identification and judgment of patients in cardiac arrest, who is suitable for CPR, CPR operation standards, the requirements of high-quality CPR, the acquisition of AEDs, the basics of AEDs and operational points, the improvement of relevant laws, and so on; in the practical part, according to the requirements of the guidelines, the following content was added: the assessment of site safety, the way of judging patients in cardiac arrest, CPR compressions, AED operation specifications, AED operation peculiarities, and simplifying the omission of mouth-to-mouth artificial respiration. The specific training model was as follows: ① training duration: 40 minutes of theory training +2.5 hours of operation training. ② Theory training: the subjects were taught intensively with lectures, which focused on the role of the first witness in emergencies, how to correctly dial the telephone for help, how to identify and judge patients with CA, and who can receive CPR, as well as its operating standards and high-quality requirements, the acquisition of an automatic external defibrillator (AED), its fundamental information and operation essentials, and the improvement of relevant laws. ③ Operation training: the teaching videos were recorded by qualified AHA trainers, including on-site safety assessments, judgment methods of CA, chest compressions, AED operation specifications, and specificity, with mouth-to-mouth resuscitation omitted. The “PPW” teaching method [11] was used to ensure the training outcome; there was one trainer and eight to ten students. The students could train on a simulator with a feedback device while watching a video. The trainer would provide the whole-course guidance and explanations to correct any problems immediately and ensure the accuracy and effectiveness of the procedure.

### 2.7. Statistical Methods

The Questionnaire Star was used for data statistics. SPSS 21.0 statistical software was used for data analysis after import. The measurement data were expressed as mean ± standard deviation $\left(\bar{X}\pm s\right)$, and the enumeration data were expressed as frequency and percentage, followed by descriptive statistics and paired *t-*tests for analysis.

## 3. Results

### 3.1. Demographics

A total of 94 residents of a community in Nantong city were selected as the subjects, including 43 males and 51 females, aged 18–29 years (*n* = 10), 30–44 years (*n* = 24), 45–59 years (*n* = 39), and over 60 years (*n* = 21); their educational background: junior high school and below (*n* = 28), senior high school (*n* = 36), undergraduate (*n* = 25), and postgraduate and above (*n* = 5). See the specific demographics in Table 1.

### 3.2. Comparison of Basic Life Support Knowledge of the Residents before and after the Training

The scores of BLS knowledge, beliefs, and behaviors of 94 community residents after training were significantly higher than before training, and the average score was 11.09 ± 4.11 and 23.42 ± 6.61, respectively, before and after the training, with differences in each item statistically significant (*P* < 0.05), as shown in Table 2.

### 3.3. Comparison of the Residents' Basic Life Support Rescue Willingness before and after the Training

The difference in the scores of the residents' BLS rescue willingness before and after the training was statistically significant (*P* < 0.05), with the scores of each item shown in Table 3.

### 3.4. Comparison of the Residents' Basic Life Support Behavior before and after the Training

The residents' average BLS behavior scores were 9.84 ± 1.756 and 13.22 ± 2.20, respectively, before and after the training. There were statistically significant differences in whether the community residents could correctly judge patient consciousness, skillfully perform BLS operations, and timely obtain an AED before and after the training (*P* < 0.05), with the scores of each item shown in Table 4.

### 3.5. Basic Life Support Process Assessment of the Residents

A total of 53 community residents (56.38%) passed the operation assessment the first time, and 41 residents (43.62%) passed it after guidance.

### 3.6. The Residents' Satisfaction with the Training Model

A total of 92.55% of the residents considered the modified BLS training model reasonable in teaching course content arrangement, 93.62% believed this model was easier for ordinary people to learn and master BLS techniques, and 91.50% felt the course arrangement met their learning needs; as shown in Table 5.

## 4. Discussion

### 4.1. Effects of the Modified Basic Life Support Training Model on the Community Residents' Knowledge and Behavior

The teaching application of the modified BLS training model was studied by the community residents in Nantong city. The study results showed that the residents' BLS knowledge scores after training were significantly higher than before training; the difference was statistically significant. Therefore, it could be concluded that skill training could improve the community residents' level of BLS knowledge. This was consistent with the study results conducted by Sun et al. [16], which demonstrated that training could improve the publics' CPR cognition and skill mastery. According to a study on BLS training among teachers by Maríía et al. [17], senior intellectuals, including teachers, could accept and learn the traditional BLS training model, but the new model targeted ordinary residents with various types of jobs and different education levels without medical knowledge; thus, the teaching content was easier for average residents to accept and master. Studies have shown [18] that it is tough for the public to obtain an AED. Although more than 40% of the residents received CPR training to some extent, their skill mastery was not ideal, and most of them did not even know the correct procedure for CPR and could not take proper rescue measures for out-of-hospital patients with CA. According to the results of this study, the behavioral ability of the community residents was significantly improved after training, especially in terms of AED acquisition ability; the differences were statistically significant before and after the training. The higher the community residents' level of BLS knowledge contributed to a more positive attitude and stronger behavioral ability.

### 4.2. The Modified Basic Life Support Training Model can Improve Community Residents' Rescue Willingness

When cardiovascular incidents occur, especially sudden death, lack of knowledge is an important factor influencing the “first witness” to provide rescue immediately [19]. Lack of knowledge makes residents fear legal liability after the rescue, which is a non-negligible situation in China [8]. Although it has been made clear that a rescuer should not bear legal responsibility for damage caused to a patient in the rescue process, relevant news reports are still common. There is still a long way to go for law popularization and BLS knowledge publicity and citizens' self-help and call-for-help awareness are still insufficient.

There were significant differences in the willingness of the public to provide rescue in emergencies before and after training, and the modified training model can greatly improve their preparedness, which may be associated with the addition of the importance of the role of the “first witness” and the “golden time of rescue” in the new BLS training model and the propaganda of relevant laws and regulations and related knowledge.

### 4.3. The Modified Basic Life Support Training Model Meets the Learning Needs of the Community Residents

According to the satisfaction survey, 91.50% of the community residents considered the course content reasonable, and the content, training pattern, and assessment met their actual needs. The combination of theory training, video teaching, and practical operation is employed in the modified BLS training model. Unlike the traditionally fragmented knowledge dissemination, in the new model, a continuous operation video was made about CPR and AEDs, and the residents conducted a practical operation in groups. During the practice, the trainers provided guidance and broke down the key points so that the public could perform the skills rather than learning the theory or watching videos only. In the opinion of 93.62% of the residents, the teaching content of the modified BLS training model made it easier for ordinary people to master, indicating that it was easier to accept than the traditional training. According to the guidelines in 2020 [5], nonprofessionals should focus on chest compression rather than mouth-to-mouth resuscitation, as incorrect artificial respiration may cause complications, such as hyperventilation, neck injury, and suffocation. Studies have shown [20] that there are no statistically significant differences in the survival and success rates of first aid between the patients receiving chest compression alone and those receiving chest compression + artificial respiration. This training omitted the “mouth-to-mouth resuscitation” step and focused on teaching using an AED and chest compressions, making it acceptable for more residents. This was consistent with the views in the guidelines in 2020 that CPR with chest compression alone was easier for the public to learn than traditional CPR (chest compression + mouth-to-mouth resuscitation). Cho and Kim [21] found that the skill accuracy of CPR training with chest compression alone was 28.5% higher than traditional training methods. Meanwhile, the omission of the “mouth-to-mouth resuscitation” step helps people pay more attention to the accuracy of the chest compressions, reduces the complications caused by improper ventilation, and dramatically reduces concerns about disease in the current challenging time of COVID-19, which increases people's willingness to help. Moreover, compared with the traditional BLS model, the modified BLS model focuses more on operational practice in terms of time and devotes most of the training practice to practice and correction. One instructor can train 8–10 trainees to ensure training quality and efficiency. In terms of instructor selection, instructors are all AHA registered ACLS instructors in the U.S. with rich teaching experience, who can design scenarios for training according to the needs of the scenario and highlight practicality.

Due to the impact of the COVID-19 pandemic, a comparison with traditional models was not conducted in this study. In addition, foreign studies have shown that people's reserve memory of first aid skills and knowledge will decline significantly within one week [22] and after six months for professionals [23], which reminds us of the need for retraining to strengthen knowledge and skills [24]. Moreover, there is evidence that retraining is beneficial to effectively preserve learning and skill memory [25], but there are no clear regulations on the interval of retraining at home and abroad. This study has not yet proposed a specific scheme of BLS retraining for the public, which will be further studied and discussed in the future.

## 5. Conclusion

The modified BLS training model could improve the community residents' rescue willingness and skill mastery rates, enhance their first-aid skills and awareness, reduce the risk of disease transmission to a certain extent, and improve the success rate of prehospital first aid to ensure the safety of rescuers and patients.

## Figures and Tables

**Table 1:** Demographics of BLS trainees (*n* = 94).

Item	Number of cases (*n*)	Constituent ratio (%)
Gender		
Male	43	45.74
Female	51	54.26
Age		
18–29 years	10	10.64
30–44 years	24	25.53
45–59 years	39	41.49
≥60 years	21	22.34
Educational background		
Junior high school and below	28	29.79
Senior high school	36	38.30
Undergraduate	25	26.60
Postgraduate and above	5	5.31
Occupation		
Peasant	12	12.77
Common employee	43	45.74
Teachers or civil servants	10	10.64
Others	29	30.85
There are patients with heart disease in your family		
Yes	32	34.04
No	62	65.96
First aid experience		
Yes	3	3.19
No	91	96.81

**Table 2:** Comparison of BLS knowledge score of residents before and after training ($\bar{x}\pm s$, scores).

Item	Before the training	After the training	*t*	*P*
Appearance of AED	0.45 ± 0.83	1.30 ± 0.96	−6.478	0.001
How to use AED	0.26 ± 0.67	1.51 ± 0.86	−11.122	0.001
When to use AED	0.32 ± 0.74	0.96 ± 1.00	−4.969	0.001
Who can receive AED	0.30 ± 0.74	0.94 ± 1.00	−5.021	0.001
Who can operate AED	0.26 ± 0.67	1.62 ± 0.79	−12.726	0.001
Who can receive CPR	0.06 ± 0.35	1.21 ± 0.98	−10.670	0.001
When to use CPR	0.36 ± 0.77	1.38 ± 0.93	−8.191	0.001
The primary measure to take when you find someone fainting	0.13 ± 0.49	1.49 ± 0.88	−13.135	0.001
How to judge respiratory arrest	1.28 ± 0.97	1.89 ± 0.45	−5.610	0.001
How to judge cardiac arrest	1.40 ± 0.92	1.85 ± 0.53	−4.086	0.001

**Table 3:** Comparison of BLS attitude scores of community residents before and after training ($\bar{x}\pm s$, scores).

Item	Before the training	After the training	*t*	*P*
Willing to provide rescue for patients	2.31 ± 0.57	2.50 ± 0.54	−2.360	0.019
Willing to provide rescue under professional guidance	2.48 ± 0.50	2.83 ± 0.57	−2.765	0.009
Willing to provide assistance after learning BLS	2.47 ± 0.50	2.71 ± 0.46	−3.503	0.001

**Table 4:** Comparison of BLS behavior scores of community residents before and after the training ($\bar{x}\pm s$, scores).

Item	Before the training	After the training	*t*	*P*
Willing to study BLS	2.60 ± 0.49	2.61 ± 0.59	−0.134	0.894
Able to correctly judge patient's consciousness	2.1 ± 0.78	2.70 ± 0.46	−5.606	0.001
Proficient in BLS operation	1.16 ± 0.37	2.63 ± 0.53	−22.101	0.001
Able to obtain AED in time	1.14 ± 0.35	2.66 ± 0.56	−22.403	0.001

**Table 5:** Evaluation of the satisfaction of community residents with modified BLS training model [*N* = 94, *n* (%)].

Items	Very satisfied	Satisfied	Neutral	Dissatisfied	Very dissatisfied
Course content	49 (52.13)	38 (40.42)	7 (7.45)	0 (0.00)	0 (0.00)
Course schedule	45 (47.88)	42 (44.68)	5 (5.32)	2 (2.12)	0 (0.00)
Meet your own needs	49 (52.14)	37 (39.36)	6 (6.38)	2 (2.12)	0 (0.00)
Easier to master	42 (44.68)	46 (48.94)	5 (5.32)	1 (1.06)	0 (0.00)
Improve the use confidence	39 (41.49)	48 (51.06)	7 (7.45)	0 (0.00)	0 (0.00)

## Data Availability

The data that support the findings of this study are available from the corresponding author upon reasonable request.
